# Supplementary material for: Complex‐centric proteome profiling by SEC‐SWATH‐MS
Source: Mol Syst Biol. 2019 Jan 14;15(1):e8438. doi: 10.15252/msb.20188438 (PMC6346213; doi:10.15252/msb.20188438)
Supplement: Supplementary file 8 — Dataset EV7 [file MSB-15-e8438-s008.zip › feature_plots_string/P49588.pdf]

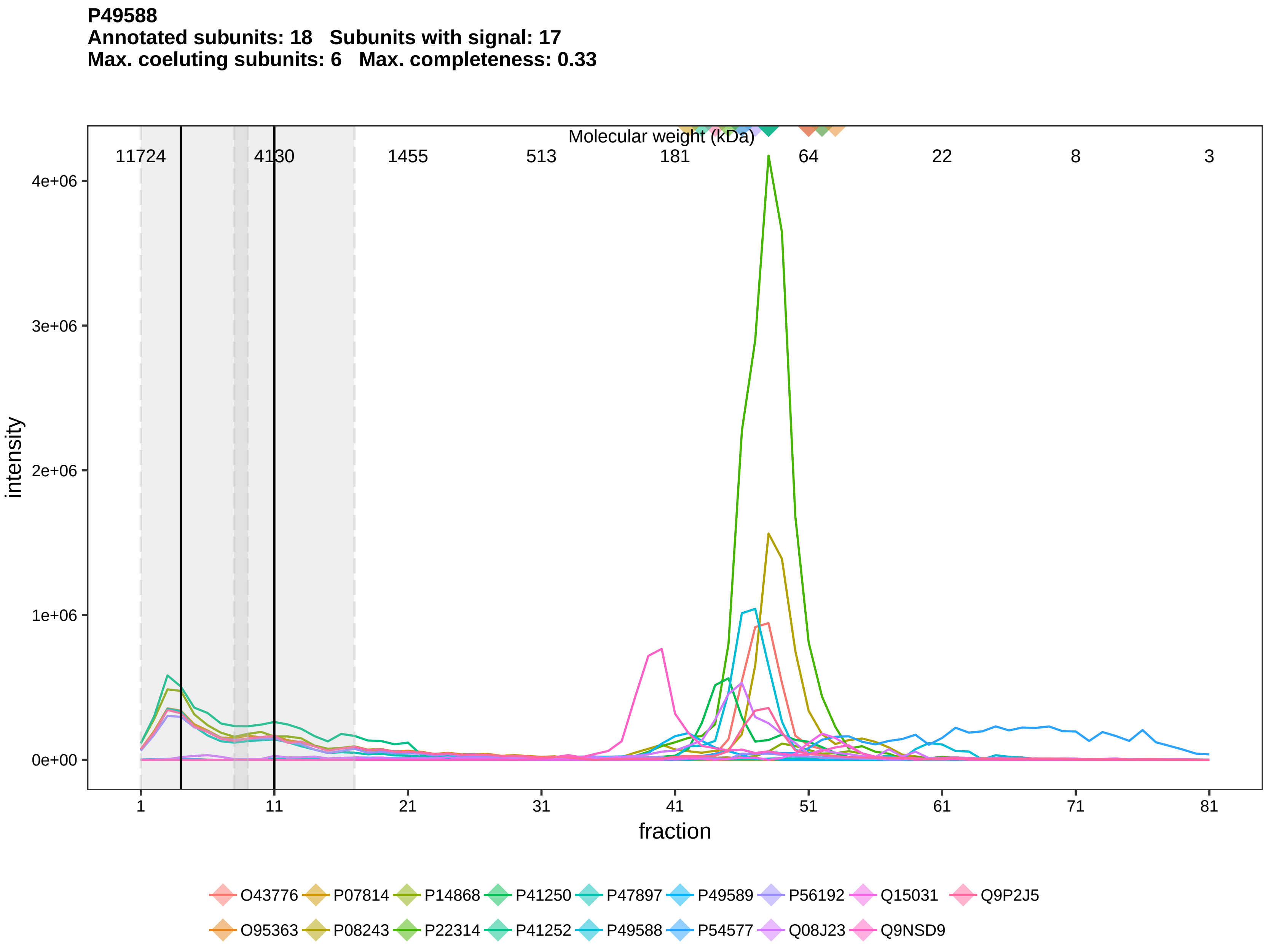

P49588
Annotated subunits: 18 Subunits with signal: 17
Max. coeluting subunits: 6 Max. completeness: 0.33
Molecular weight (kDa)
11724
4130
1455
513
181
64
22
8
3
4e+06
3e+06
2e+06
1e+06
0e+00
intensity
1
11
21
31
41
51
61
71
81
fraction
O43776
P07814
P14868
P41250
P47897
P49589
P56192
Q15031
Q9P2J5
O95363
P08243
P22314
P41252
P49588
P54577
Q08J23
Q9NSD9
